# The Phenotype of Elderly Patients with Type 2 Diabetes Mellitus and Poor Sleep Quality

**DOI:** 10.3390/ijerph17165992

**Published:** 2020-08-18

**Authors:** Melania Mikołajczyk-Solińska, Agnieszka Śliwińska, Marcin Kosmalski, Józef Drzewoski

**Affiliations:** 1 Department of Internal Medicine, Diabetology and Clinical Pharmacology, Medical University of Lodz, 92-213 Lodz, Poland; 2 Department of Nucleic Acids Biochemistry, Medical University of Lodz, 92-213 Lodz, Poland; agnieszka.sliwinska@umed.lodz.pl; 3 Department of Clinical Pharmacology, Medical University of Lodz, 90-153 Lodz, Poland; marcin.kosmalski@umed.lodz.pl; 4 Central Teaching Hospital of Medical University of Lodz, 92-213 Lodz, Poland; jozef.drzewoski@umed.lodz.pl

**Keywords:** the elderly, Type 2 diabetes mellitus, sleep quality, Pittsburgh Sleep Quality Index, hypnotics

## Abstract

Background: Sleep disturbances are a common problem among patients with Type 2 diabetes mellitus (T2DM). The aim of the study was to identify the phenotype of T2DM patients with poor sleep quality. Methods: An observational, cross-sectional study was conducted between May 2013 and August 2015. One hundred and sixty consecutive patients with T2DM: 74 women and 86 men, with a median age of 69.50 years (59.00; 79.50 years) were enrolled in the study. Sleep quality was evaluated using the Pittsburgh Sleep Quality Index (PSQI) questionnaire. Results: Poor sleep quality was noted in 85 (53%) patients. Sleep disorders were associated with older age, as well as female gender, longer duration of diabetes, lower level of fasting plasma glucose, glycated hemoglobin A1c, estimated glomerular filtration rate, triglycerides, waist-to-hip ratio, and the presence of nephropathy. A multivariate logistic regression revealed that sleep disorders were associated with older age (Odd Ratio (OR) = 1.11, 95% Confidence Interval (CI) 1.07–1.15). Fifty-one patients (31.87%) were treated with sleeping pills. We found that older age, female gender, longer duration of diabetes, lower level of fasting plasma glucose, glycated hemoglobin A1c, estimated glomerular filtration rate, triglycerides, and the presence of nephropathy were linked with more frequent usage of hypnotics. A multivariate logistic regression demonstrated that older age (OR = 1.09, 95% CI 1.05–1.14) and nephropathy (OR = 2.79, 95% CI 1.24–6.28) were associated with a more frequent receiving the hypnotics, whereas male gender (OR = 0.30, 95% CI 0.13–0.71) has less frequent hypnotics usage. Conclusion: Although, we assessed a wide range of patients’ characteristics, age had the most negative impact on the quality of sleep in patients with T2DM. We detected more frequent use of hypnotics in older females, with coexisting nephropathy.

## 1. Introduction

Sleep disorders are common problem nowadays. Modern society, characterized by widespread electricity use, modern technology, shift work, prolonged commute times and social activities, has significantly changed our sleep patterns [1,2]. The average self-reported sleep duration has decreased from over 8 hours in the 1960s to 6.5 h in 2012 [3]. Problems with sleeping affect more frequent the elderly. Word Health Organization recognizes the beginning of old age at the age of 60 [4]. However, the United Nations and most developed world countries have accepted the chronological age of 65 years as a definition of elderly [5].

Elderly patients often present primary sleep disorders such as sleep apnea syndrome, restless legs syndrome, and rapid eye movement [6]. In addition to primary sleep disorders, it is well known that there are many factors responsible for worsening quality of sleep in the elderly, including aging, chronic diseases, medications, and bad sleep habits [7]. It is believed that stimulants and environmental factors can also disturb the quality of sleep [8,9]. Sleep problems have serious consequences and lead to abnormal physical, mental, social, and emotional functioning [10].

Epidemiological data suggest that the prevalence of various sleep disorders in the general population ranges from 0.047% to 50.5%, being the highest in the elderly [11,12]. Studies reported the presence of sleep disorders in diabetic patients is higher, ranged approximately between 50–70% [13]. The estimated number of people over 65 years of age with diabetes worldwide is 111 million, and it is raising [14].

Patients suffering from Type 2 diabetes mellitus (T2DM) exhibit phenotype features predisposing to deterioration of sleep such as insulin resistance related obesity [15] and sleep disordered breathing [16], peripheral neuropathy [17], restless leg syndrome [18], and nocturnal hypoglycemia [19].

The growing number of medical evidences indicate that the abnormal quantity and poor quality of sleep have a negative impact on glucose metabolism [20,21]. Clinical observations indicate that diabetes, both Type 1 and Type 2 favor sleep disorders and insomnia [22,23]. What is more, diabetes related sleep disorders have a negative impact on glycemic control [24,25] and increase the risk of developing chronic diabetic complications [26,27]. The studies conducted in patients with T2DM have shown that the abnormal duration of sleep, both too short and too long (U-curve relationship), increase the risk of cardiovascular events and death for any reason [28,29,30]. Furthermore, diabetic complications both micro- and macroangiopathic have negative impact on the quality and quantity of sleep [31,32].

It has been suggested that there is association between sleep quality and the type of older generation of hypoglycemic drugs. Barakat et al. found that poor sleep quality in patients with T2DM is significantly associated with insulin use [33]. In turn, Kajbaf et al. reported that metformin therapy in patients with T2DM is related to longer sleep duration and better sleep efficiency. There is limited evidence connected to newer antidiabetic drugs and sleep quality in patients with T2DM [34].

Although sleep abnormalities are receiving plenty of attention, there is relatively little data regarding the association between anthropometric characteristics, biochemical parameters, chronic T2DM complications, and sleep disorders. Therefore, the purpose of this study was to characterize the phenotype of T2DM patients with poor sleep quality.

## 2. Materials and Methods

The study protocol was approved by the Bioethics Committee of the Medical University of Lodz (decision no. RN/106/13/KE). The study was performed in accordance with all relevant ethical principles of the Declaration of Helsinki. All patients provided written informed consent to participate in the study.

An observational, cross-sectional study was conducted between May 2013 and August 2015, in the Department of Internal Medicine, Diabetology, and Clinical Pharmacology Medical University of Lodz, Poland. The study enrolled 160 consecutive hospitalized patients with T2DM: 74 women and 86 men, with a median age of 69.50 years (59.00; 79.50 years). All participants were ethnic homogenic, namely representatives of Caucasian from region of Lodz in Poland. The inclusion criteria for the study were as follows: the age over 18 and T2DM diagnosed on the basis of medical history and/or taking hypoglycemic medications. Exclusion criteria were primary sleep disorders (insomnia, sleep-related breathing disorders such as obstructive sleep apnea syndrome, hypersomnia, disturbances of the circadian rhythm, parasomnia, movement disorders related to sleep such as restless legs syndrome), psychiatric disorders (as well as drugs used to treat depression, epilepsy, and psychotic disorders), somatic diseases (common in patients with T2DM and that could interfere with quality of sleep and glucose control parameters such as thyroid disorders, anemia, and iron deficiency), and limited verbal-logical contact to understand or provide informed written consent. According to the design of study all patients, who received and completed the Pittsburgh Sleep Quality Index (PSQI) questionnaire were divided into two groups: good (sound) and poor sleepers in depending on the PSQI result. Medical history, including information on duration of the T2DM and its complications was taken from each patient. A thorough physical examination was also carried out. Each patient was subjected to an ophthalmologic examination for the presence of diabetic changes in the retina. A neurological examination was performed in order to confirm or rule out peripheral neuropathy based on the Neuropathy Disability Score (NDS).

### 2.1. Anthropometric and Biochemical Measurements

The height, body mass, waist, and hip circumference were measured in the morning after an overnight fast. The Body Mass Index (BMI) was calculated as body weight (kilograms) divided by the square of body height (meters) [35] and the Waist/Hip Ratio (WHR) was calculated as waist measurement divided by hip measurement (centimeters) [36]. A venous blood sample (9 mL) was collected from each patient in the morning after at least eight hours of overnight fasting. Serum level of glucose (Fasting Plasma Glucose (FPG)), Glycated Hemoglobin A1c (HbA1c), creatinine, Total Cholesterol (TC), High-Density Lipoprotein (HDL), Low-Density Lipoprotein (LDL), Triglycerides (TG), the Activity of Alanine Aminotransferase (ALT), and Aspartate Aminotransferase (AST) were assessed. To assess selected laboratory parameters the following methods were used: FPG—enzymatic method with hexokinase; HbA1c—immunoturbidimetric method; creatinine, TC, HDL, LDL, and TG—enzymatic-colorimetric method; and ALT and AST—enzymatic method. The estimated Glomerular Filtration Rate (eGFR) was calculated based on the Chronic Kidney Disease Epidemiology Collaboration (CKD-EPI) formula [37].

### 2.2. Evaluation of the Quality of Sleep

The patients were asked to answer the questions contained in the Pittsburgh Sleep Quality Index (PSQI) questionnaire. It consisted of nineteen individual items, creating seven components of sleep and one global score. The total score possible to obtain ranged from 0 to 21 points. If the sum of points was ≤ 5, the quality of sleep was defined as good. However, the value exceeding 5 points indicated the poor quality of sleep. The reliability of the Pittsburgh Sleep Quality Index (PSQI) scale is recognized as satisfactory with Cronbach’s alpha of 0.83 for the total score. Test–retest reliability is considered as sufficient. The validity of PSQI has been described with a sensitivity of 89.6% and a specificity of 86.5% of patients versus control subjects [38].

### 2.3. Statistical Analysis

Quantitative variables were expressed as mean and standard deviation in the case of normal distribution or as median and the first (Q1) and third (Q3) quartiles in the case of non-normal distribution. Categorical variables were demonstrated as the number of observations with a given variant (n) and the corresponding percentage (structure indicator) for each variable category. Distribution of quantitative variables was assessed by the Shapiro–Wilk test. To compare the values of quantitative variables, the following tests were used: the Student’s t-test for normal distribution of the variables tested; the Levene’s test for homogeneity of variance; and the non-parametric U Mann–Whitney test with correction for rank for non-normal distribution of even one variable. Due to the large sample size, the Z statistic was used. To examine differences between proportions for independent groups, the Z test was used [39].

To measure the effect size, we used Cohen’s d (d). d < 0.2 meant no effect size, d ≥ 0.2 was a “small” effect size, d ≥ 0.5 represented a “medium” effect size and d ≥ 0.8 a “large” effect size [40].

Multivariate analysis of clinical and biochemical parameters was performed using logistic regression analysis. The backward elimination method was used to select predictive variables. The linear nature of dependence between included variable and each predictor was checked using the Likelihood Ratio test (LR test). The goodness of fit was evaluated by the LR test and the Hosmer–Lemeshow test. The significance of logistic coefficients was examined with the Wald’s test. The model prediction quality was evaluated by the ten-fold cross-validation method and the subsequent Receiver Operating Characteristic (ROC) curve with an Area Under the Curve (AUC) as a quality index. The analyses were conducted using the STATISTICA PL v. 7.1. statistical package (Statsoft, Tulsa, OK, USA) and PQSTAT (PQSTAT Software Company, Poznan, Poland). The *p* value less than 0.05 was considered statistically significant.

## 3. Results

The study included 160 patients with T2DM. There were 39 patients who received insulin in monotherapy, 35 insulin plus oral antidiabetic agents (metformin and/or sulphonylurea and/or acarbose), 76 oral antidiabetic agents, and 10 were treated only with diet. The median PSQI score for all patients with T2DM was 6.00 (3.00; 11.50). The percentage of poor sleepers among patients with T2DM was 53% (PSQI > 5).

To identify the phenotype of patients with T2DM and sleep problems, we performed further analysis covering anthropometric and biochemical parameters, glucose control parameters, and coexisting diabetic complications by comparing poor and sound sleepers. Comparative clinical characteristics are shown in Table 1. Poor and sound sleepers did not differ in relation the following parameters: BMI, systolic blood pressure, diastolic blood pressure, level of TCH, LDL, HDL, ALT, AST, presence of retinopathy, peripheral neuropathy, myocardial infarction, and stroke. We found that poor sleepers were older, had longer duration of diabetes, significantly lower level of FPG, HbA1c, GFR, TG, and WHR. Our observations indicate that women with T2DM reported worse quality of sleep than men. Nephropathy was significantly more frequent in patients with T2DM and sleep disorders. For all evaluated clinical parameters, the strongest effect size was found for age (d = 1.31).

**Figure 1:**
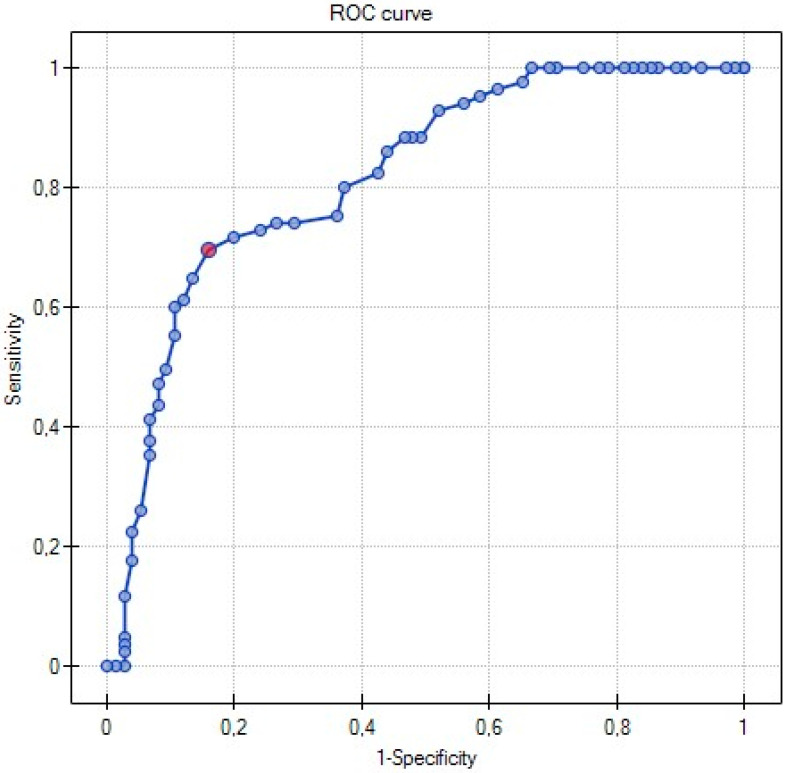
Receiver operating characteristic (ROC) presents prognosis for sleep problems in aging patients with T2DM. AUC, area under the curve.

Multivariate logistic regression analysis was used to examine the impact of tested variables on sleep quality in diabetics. It revealed that aging was a key risk factor for poor quality of sleep in T2DM patients (Odd Ratio (OR) = 1.11, 95% Confidence Interval (CI) 1.07–1.15) (Table 2). Moreover, the ROC analysis revealed that age differentiates the quality of sleep. T2DM patients over the age of 72 had 12 times more chance for developing sleep problems than younger patients (Area Under the Curve (AUC) = 0.81, Standard Error (SE) = 0.03) (shown in Figure 1).

Fifty-one patients with T2DM (31.87%) were treated with hypnotics including prescription drugs, over-the-counter drugs, and herbal medicines. The most commonly used drugs were hydroxyzine, benzodiazepines, and Z drugs (zolpidem, zopiclone, and zaleplon) (Table 3). Due to the fact that one-third of patients with T2DM were treated with hypnotics it prompted us to evaluate the phenotype of a diabetic patient using sleeping pills. Users and non-users of hypnotics did not differ in relation the following parameters: BMI, systolic blood pressure, diastolic blood pressure, level of TCH, LDL, HDL, TG, ALT, AST, presence of retinopathy, peripheral neuropathy, myocardial infarction, and stroke. We found that patients with T2DM using hypnotics were older, had longer duration of diabetes, and had lower GFR, TG, and HbA1c levels. Women used sleeping pills three times often than men (74.51% vs. 25.49%). We observed that nephropathy was significantly more common in patients with T2DM treated with hypnotics. For all evaluated clinical characteristics between users and non-users of sleeping pills, the strongest effect size was found for the male gender (OR = 0.26, 95% CI 0.12–0.56), age (d = 1.03), and nephropathy (OR = 3.68, 95% CI 1.80–7.50) (Table 4).

Multivariate logistic regression analysis was used to examine the impact of tested variables on the usage of hypnotics among patients with T2DM. We reported that both aging (OR = 1.09, 95% CI 1.05–1.14) and the presence of nephropathy (OR = 2.79, 95% CI 1.24–6.28) were related to more common hypnotics treatment. The male gender was associated with less frequent usage of sleeping pills by diabetic patients (OR = 0.3, 95% CI 0.13–0.71) (Table 5).

## 4. Discussion

In our study, 53% of patients with T2DM were poor sleepers, which is consistent with the observations of other authors. Keskin et al. revealed 64.3% patients with T2DM (585 subjects, median age 57 (50–64 years)) experienced poor-quality sleep [41]. Lecube et al. conducted a study including 413 patients with T2DM (mean age 55.9 (10.4)), and 413 non-diabetic subjects (mean age 55.0 (10.0)), matched by age, gender, BMI, and waist and neck circumferences. They found that sleep disturbances are more common in subjects with T2DM (PSQI: 7.0 (1.0–18.0) vs. 4 (0.0–12.0), *p* < 0.001). Moreover, 67.4% of T2DM patients were classified as poor sleepers [42].

Evaluating the phenotype of patients with T2DM and sleep problems we found that advanced age is one of the most important risk factors promoting sleep dysregulation. To our best knowledge our study is one of the few evaluating sleep quality among elderly patients with T2DM. It should be stresses that patients included to our study were older than subjects in cited research [41,42]. We also found that sleep problems were significantly more frequent in patients with longer duration of T2DM. These observations are consistent with the results of Keskin research that duration of diabetes is positively correlated with PSQI scores [41]. Moreover, we revealed the worse quality of sleep in women with T2DM. Nefs at al. also documented that poor sleep quality in patients with T2DM was associated with female gender (OR = 2.72, CI 1.42–5.20) [43].

Interestingly, we reported that patients with T2DM and poor sleep quality have significantly lower WHR. This is contrary to other studies that documented sleep disorders, mainly sleep apnea in patients with abdominal obesity [44]. However, it should be emphasized that the elderly, as well as studied group, display body weight loss and sarcopenia [45].

It was found in presented study that patients with T2DM and poor quality of sleep had significantly lower values of diabetes metabolic control parameters. This is in contrast to observations of other authors, who documented the correlation between poor quality of sleep and higher level of FPG and HbA1c [24,25]. It was demonstrated that sleep duration below 6 hours and above 9 hours per day had link with higher level of FPG and HbA1c. This suggests the U-curve relationship between sleep duration and the level of FPG and HbA1c in patients with T2DM [28]. Interestingly, Kim et al. found that in the elderly (≥65 years), a short sleep duration (6 hours per day) was associated with the lowest HbA1c (7.26%) [46]. It is in accordance with our observations, that the average age of patients with T2DM and poor quality of sleep was 79 years and the mean value of HbA1c was 7.28%. Our findings could suggest that some of the elderly patients with T2DM might be overtreated. Tight glycemic control and use of diabetes medications, especially insulin and sulphonylureas, could result in hypoglycemia [47]. The short- and long-term complications of diabetes related to hypoglycemia include cardiac arrhythmias, myocardial infarction, neurocognitive dysfunction, serious falls, frailty, and death [48,49,50].

In our study we did not find the relationship between poor sleep quality in patients with T2DM and retinopathy, neuropathy, myocardial infarction, and stroke. However, we noted reduced GFR and increased frequency of nephropathy. Ahmad et al. demonstrated that as many as one-third of patients with chronic kidney disease complained of insomnia [51]. Poor sleep quality in patients with chronic kidney disease may be related to metabolic changes such as fluid and electrolytes imbalance, anamia, and hypoalbuminemia. The sleeping problems in this group also correlate with comorbidities, mainly cardiovascular and liver diseases. In patients with end stage renal disease and on dialysis, sleep deterioration is also strongly associated with psychological disturbances like anxiety and depression [52].

It should be recognized that other confounding factors, which were not evaluated in our study, could worsen the quality of sleep in patients with T2DM in our study. They include nocturia, comorbidities such as heart failure, asthma, chronic obstructive pulmonary disease, gastroesophageal reflux disease, arthritis, and medications, mainly diuretics and stimulants.

There is limited data about pharmacotherapy of sleep disorders in patients with T2DM. The results of our study demonstrated that one-third of patients with T2DM were treated with hypnotics. The most frequently applied drugs were hydroxyzine, benzodiazepines, zopiclone, zolpidem, and zaleplon, whereas melatonin and herbal preparations were used less frequently. Lin et al. documented that the use of benzodiazepines and zolpidem significantly increase the risk of T2DM [53]. Moreover, there is an evidence that chronic use of hypnotics, especially benzodiazepines, could have a negative impact on patients through potentiate memory disturbance, increase number of falls, and reduce life expectancy [54,55,56].

We found that the frequency of taking hypnotics increased with age and the duration of T2DM. Kaufman et al. observed that most patients using hypnotics were patients over 65 years [57]. There is no data whether the same statistics are in diabetics. Our research also revealed that women with T2DM use hypnotics more often than men. It is in accordance with Swedish study showing that women are more likely than men to use all prescribed medications [58]. It was found in the present study that patients with T2DM receiving hypnotics demonstrated a lower level of HbA1c. As we have already mentioned, lower values of this diabetes control marker could reflect the episodes of hypoglycemia and promote sleep problems [48,49,50].

In our research patients with lower GFR and diabetic kidney disease were treated with hypnotics more often than subjects with proper functioning of this organs. Plantinga et al. reported that frequency of sleep problems and using sleeping pills increases with stage of chronic kidney disease. They reported that the percentage of patients treated with hypnotics in stage 1 and 2 was 8.4%, in stage 3—9.9% and in stage 4—16.6% [59]. We did not find any relationship between the frequency of hypnotic’s usage and other micro- and macrovascular complications of diabetes.

There are common risk factors for sleep disorders in general population and in patients with T2DM, such as aging and female gender. However, in patients with T2DM more attention should be paid to the elderly with longer duration of the disease, lower anthropometric parameters, decreased levels of glucose and lipids metabolism markers, and with presence of microvascular complications.

The main strength of our study is presentation the phenotype of patient with T2DM at high risk of poor sleep quality in order to quickly recognize this subject and implement behavioral or pharmacotherapy treatment. Furthermore, we focused on the important and alarming medical issue, namely the growing number of people in older age with diabetes suffering from sleep disorders. Moreover, the study group consisted of the elderly who are often not included in clinical studies. To our best knowledge, this is the first study investigating the pharmacotherapy of sleep disorders in the elderly patients with T2DM. The following limitations of the study should be recognized: relatively small study group and PSQI results were influenced by hospitalization. Confounding factors such as nocturia, comorbidities such as heart failure, asthma, chronic obstructive pulmonary disease, gastroesophageal reflux disease, arthritis, and medications, mainly diuretics and stimulants; however, they were not investigated.

## 5. Conclusions

Although, we assessed a wide range of patients’ characteristics, age had the most negative impact on the quality of sleep in patients with T2DM similarly to general population. We also found, that the longer duration of diabetes, female gender, lower level of FPG, HbA1c GFR, and TG, as well as WHR and presence of nephropathy, were significantly associated with poor quality of sleep in patients with T2DM. Similar parameters, including older age, longer duration of diabetes, lower level of FPG, HbA1c, GFR, and TG affected significantly the hypnotic usage. The strongest relationship with more frequent use of hypnotics we detected in older females, with coexisting nephropathy.

In the face of a major epidemiological problem, clinicians should search for sleep problems in patients with T2DM, especially in the elderly. Further studies are needed to detect if early detection of the sleep problems and appropriate behavioral or pharmaceutical interventions could improve patients’ quality of life and diabetes treatment results.

## Figures and Tables

**Table 1:** Phenotype of poor and good sleepers among T2DM patients.

Variable	Poor Sleepers (PSQI >5) *n* = 85	Good Sleepers (PSQI ≤5) *n* = 75	*p*	Effect Size (Cohen’s d/OR)
Gender (male/female)	29/56	45/30	0.001	OR 0.34 95% CI 0.18–0.65
Age (years)	79.00 (71.00; 84.00)	60.04 (13.54)	<0.001	1.31
Diabetes duration (years)	5.00 (2.00; 12.00)	2.00 (1.00; 5.00)	<0.001	0.71
BMI (kg/m²)	29.17 (25.81; 32.77)	28.68 (25.86; 33.45)	0.97	0.006
WHR	0.94 (0.90; 1.00)	0.99 (0.08)	0.006	0.51
Systolic blood pressure (mm Hg)	140.00 (125.00; 150.00)	130.00 (120.00; 150.00)	0.23	0.18
Diastolic blood pressure (mm Hg)	80.00 (70.00; 90.00)	80.00 (70.00; 90.00)	0.93	0.01
eGFR (ml/min/1.73m²)	65.00 (43.00; 88.80)	74.10 (51.30; 106.30)	0.04	0.32
TCH (mmol/l)	4.25 (1.25)	4.58 (1.36)	0.10	0.26
LDL (mmol/l)	2.45 (1.69; 3.16)	2.72 (2.04; 3.53)	0.06	0.29
HDL (mmol/l)	1.08 (0.89; 1.36)	1.01 (0.77; 1.28)	0.09	0.26
TG (mmol/l)	1.34 (0.99; 1.84)	1.88 (1.11; 2.74)	0.001	0.52
ALT (U/l)	21.00 (14.00; 31.00)	23.00 (16.18; 38.00)	0.20	0.08
AST (U/l)	21.00 (18.00; 28.00)	20.00 (15,00; 35.00)	0.69	0.04
FPG (mmol/l)	6.41 (5.68; 7.90)	7.10 (5.99; 8.50)	0.02	0.42
HbA1c (%)	7.28 (6.24; 8.72)	8.40 (6.90; 10.39)	0.004	0.46
Nephropathy (*n*,%)	33 (38.82%)	17 (22.66%)	0.02	OR 2.16 95% CI 1.08–4.33
Retinopathy (*n*,%)	11 (12.94%)	9 (12.00%)	0.85	OR 1.09 95% CI 0.42–2.79
Peripheral neuropathy (*n*,%)	10 (11.76%)	8 (10.66%)	0.82	OR 1.11 95% CI 0.41–2.99
Miocardial infarction (*n*,%)	15 (17.64%)	12 (16.00%)	0.78	OR 1.12 95% CI 0.49–2.15
Stroke (*n*,%)	14 (16.47%)	13 (17.33%)	0.88	OR 0.94 95% CI 0.41–2.15

Data is presented as mean (SD) or median (lower and upper quartiles); *p* value < 0.05 difference statistically significant, ALT, Alanine Aminotransferase; AST, Aspartate Aminotransferase; BMI, Body Mass Index; CI, Confidence Interval; FPG, Fasting Plasma Glucose; eGFR, Estimated Glomerular Filtration Rate; HbA1c, Glycated Hemoglobin A1c; HDL, High-Density Lipoprotein; LDL, Low-Density Lipoprotein; OR, Odds Ratio; PSQI, Pittsburgh Sleep Quality Index; T2DM, Type 2 Diabetes Mellitus; TC, Total Cholesterol; TG, Triglycerides; and WHR, Waist-to-Hip Ratio.

**Table 2:** Multivariate logistic regression analysis for poor sleep in patients with T2DM.

Variable	Estimates	SE	Wald’s *p*	OR	−95% CI	+95% CI
Age	0.11	0.01	<0.001	1.11	1.07	1.15

Hosmer–Lemeshow test value = 6.90, *p* = 0.54; CI, Confidence Interval; OR, Odds Ratio; and SE, Standard Error.

**Table 3:** Number and type of hypnotics taken by patients with T2DM.

Type of Hypnotics	T2DM Patients *n* = 160
Benzodiazepines	16 (10%)
Hydroxyzine	19 (11.87%)
Z drugs (zolpidem, zopiclone, zaleplon)	10 (6.25%)
Melatonin	3 (1.87%)
Herbal medicines	3 (1.87%)
Total	51 (31.87%)

T2DM, Type 2 Diabetes Mellitus.

**Table 4:** Phenotype of users and non-users of hypnotics among T2DM patients.

Variable	Patients with T2DM Using Hypnotics *n* = 51	Patients with T2DM Not Using Hypnotics *n* = 109	*p*	Effect Size (Cohen’s d/OR)
Gender (male/female)	13/38	61/48	<0.001	OR 0.26 95% CI 0.12–0.56
Age (years)	80.00 (72.00; 84.00)	63.90 (13.75)	<0.001	1.03
Diabetes duration (years)	8.00 (2.00; 13.00)	2.00 (1.00; 6.00)	<0.001	0.63
BMI (kg/m²)	30.14 (5.04)	28.37 (25.39; 33.07)	0.34	0.17
WHR	0.95 (0.91; 1.00)	0.97 (0.08)	0.19	0.25
Systolic blood pressure (mm Hg)	140.00 (120.00; 150.00)	130.00 (120.00; 150.00)	0.55	0.09
Diastolic blood pressure (mm Hg)	80.00 (70.00; 90.00)	80.00 (70.00; 90.00)	0.61	0.08
eGFR (ml/min/1.73m²)	60.00 (39.00; 85.20)	73.00 (54.00; 102.00)	0.003	0.47
TCH (mmol/l)	4.22 (3.11; 4.99)	4.30 (3.68; 5.33)	0.11	0.24
LDL (mmol/l)	2.37 (1.63; 3.17)	2.66 (2.04; 3.39)	0.08	0.27
HDL (mmol/l)	1.10 (0.88; 1.33)	1.03 (0.81; 1.31)	0.29	0.16
TG (mmol/l)	1.30 (0.94; 1.84)	1.64 (1.06; 2.58)	0.01	0.40
ALT (U/l)	21.00 (14.00; 31.00)	23.00 (16.00; 36.00)	0.25	0.18
AST (U/l)	24.00 (18.00; 28.00)	20.00 (16.0; 29.00)	0.32	0.24
FPG (mmol/l)	6.31 (5.65; 7.79)	7.02 (5.98; 8.17)	0.05	0.30
HbA1c (%)	7.13 (6.17; 8.41)	8.20 (6.71; 9.91)	0.004	0.46
Nephropathy (*n*, %)	26 (50.98%)	24 (22.01%)	<0.001	OR 3.68 95% CI 1.80–7.50
Retinopathy (*n*, %)	6 (11.76%)	14 (12.84%)	0.84	OR 0.90 95% CI 0.32–2.50
Peripheral neuropathy (*n*, %)	5 (9.80%)	13 (11.92%)	0.69	OR 0.80 95% CI 0.27–2.38
Miocardial infarction (*n*, %)	9 (17.64%)	18 (16.51%)	0.85	OR 1.08 95% CI 0.44–2.61
Stroke (*n*, %)	7 (13.72%)	20 (18.34%)	0.46	OR 0.70 95% CI 0.27–1.80

Data is presented as mean (SD) or median (lower and upper quartiles); *p* value < 0.05 difference statistically significant, ALT, Alanine Aminotransferase; AST, Aspartate Aminotransferase; BMI, Body Mass Index; CI, Confidence Interval; FPG, Fasting Plasma Glucose; eGFR, Estimated Glomerular Filtration Rate; HbA1c, Glycated Hemoglobin A1c; HDL, High-Density Lipoprotein; LDL, Low-Density Lipoprotein; OR, Odds Ratio; PSQI, Pittsburgh Sleep Quality Index; T2DM, Type 2 Diabetes Mellitus; TC, Total Cholesterol; TG, Triglycerides; and WHR, Waist-to-Hip Ratio.

**Table 5:** Multivariate logistic regression analysis for taking hypnotics by T2DM patients.

Variable	Estimates	SE	Wald’s *p*	OR	−95% CI	+95% CI
Age	0.09	0.02	<0.001	1.09	1.05	1.14
Gender (male/female)	−1.18	0.42	0.005	0.30	0.13	0.71
Nephropathy	1.02	0.41	0.01	2.79	1.24	6.28

Hosmer–Lemeshow test value = 3.64, *p* = 0.88; CI, Confidence Interval; OR, Odds Ratio; and SE, Standard Error.
